# Chemical composition and antimicrobial activity of *Cymbopogon nardus* citronella essential oil against systemic bacteria of aquatic animals

**Published:** 2013-06

**Authors:** Lee Seong Wei, Wendy Wee

**Affiliations:** 1 Faculty of Agro Based Industry, University of Malaysia, Kelantan Jeli Campus, 17600 Jeli, Kelantan, Malaysia; 2 Department of Fisheries Science, Faculty of Fisheries and Aqua-Industry, Universiti Malaysia Terengganu, 21030 Kuala Terengganu, Terengganu, Malaysia

**Keywords:** citronella essential oil, *Cymbopogon nardus*, fish systemic bacteria, aquaculture

## Abstract

**Background & Objectives:**

This paper describes chemical composition and antimicrobial activity of *Cymbopogon nardus* citronella essential oil against *Edwardsiella* spp. (n = 21), *Vibrio* spp. (n = 6), *Aeromonas* spp. (n = 2), *Escherichia coli* (n = 2), *Salmonella* spp. (n = 2), *Flavobacterium* spp. (n = 1), *Pseudomonas* spp. (n = 1) and *Streptococcus* spp. (n = 1) isolated from internal organs of aquatic animals. Due to the ban of antibiotics for aquaculture use, this study was carried out to evaluate the potential of citronella essential oil as alternative to commercial antibiotic use against systemic bacteria in cultured aquatic animals.

**Materials & Methods:**

The essential oil of *C. nardus* was prepared by using the steam distillation method and the chemical composition of the essential oil was analyzed by gas chromatography–mass spectroscopy (GC–MS). Minimum inhibitory concentration (MIC) of the essential oil tested against bacterial isolates from various aquatic animals and ATCC type strains were determined using two-fold broth micro dilution method with kanamycin and eugenol as positive controls.

**Results:**

A total of 22 chemical compounds were detected in *C. nardus* essential oil with 6-octenal, 3, 7-dimethyl- or citronellal representing the major compounds (29.6%). The MIC values of the citronella oil ranged from 0.244 µg/ml to 0.977 µg/ml when tested against the bacterial isolates.

**Conclusion:**

The results of the present study revealed the potential of *C. nardus* essential oil as alternative to commercial antibiotics for aquaculture use.

## INTRODUCTION

The intensification of aquaculture and globalization of the seafood trade have led to remarkable developments in the aquaculture industry. However, disease outbreak is always a drawback to the development of aquaculture with estimated losses around USD 3 billion per year (1, 2). Bacterial disease is one of the diseases that posed a threat to the aquaculture industry and use of antibiotics is a popular solution to bacterial infections (2). Although most antibiotics have been banned for aquaculture use, farmers were left with no option but to continue using antibiotics illegally to save their crops from devastation. For instance, it was reported that 56 out of 76 shrimp farmers in Thailand used antibiotics such as chloramphenicol, gentamycin, trimethoprim, tiamulin, tetracycline, quinolones and sulfonamides for prophylactic purposes (3). This has in turn increased shrimp production to 600 tonnes in 1994. (4). Likewise, chemotherapy is also widely practiced in aquaculture in Philippines, where most commonly used antibiotics are oxytetracycline, oxolinic acid, chloramphenicol, furazolidone, nitrofurans, erythromycin and sulfa drugs (5). However, residues from misuse and overuse of antibiotics application could be a serious problem by developing antibiotic resistance among bacteria, where the aquatic environment could become a potential reservoir for dissemination of resistant genes to humans and animals (6, 7).

Citronella or *Cymbopogon nardus* is one of the *Cymbopogon* species with its essential oil widely used in the production of citronella essential oil, food, drink, perfumery, soap, body care products and pharmaceutical products. Many studies have reported on the antifungal and antimicrobial property of *C. nardus* essential oil. Billerbeck *et al*. (2001) claimed that essential oil of *C. nardus* at concentration of 400 mg/L could inhibit 80% of *Aspergillus niger* growth (8). Meanwhile, Oussalah *et al*. (2006) reported that the essential oil showed antimicrobial activity at concentration of 4 mg/mL against *Pseudomonas putida* CRDAV 372 isolated from fresh beef (9). However, until now, no study has been conducted to investigate the antimicrobial property of *C. nardus* essential oil against causative agents of bacterial diseases in cultured aquatic animals. Therefore, this study was carried out to reveal the chemical composition and potential of *C. nardus* essential oil as an alternative to commercial antibiotics for aquaculture use.

## MATERIALS AND METHODS

### *Cymbopogon nardus* essential oil preparation

In the present study, fresh *C. nardus* was purchased from wet market in Kelantan, Malaysia. The plant sample was then air dried to 30% of the fresh weight. Aerial part of the plant sample was cut into small pieces and subjected to 3 hours of steam distillation. Essential oil was extracted at 3.0% (wt/vol) of the dried sample and stored in the dark at 4°C until further use (10).

### Identification of chemical compound in *Cymbopogon nardus* essential oil

The chromatographic procedure was carried out using Shimadzu QP2010-GC-MS with autosampler (13). The sample was diluted 25 times with acetone and 1 µl of the sample was injected into a column. A fused silica capillary column HP5-MS (30 m × 0.32 mm, film thickness 0.25 µm) was used. Helium was the carrier gas and a split ratio of 1/100 was used. The oven temperature was maintained at 60°C for 8 min and gradually raised at a rate of 3°C per min to 180°C and maintained at 180°C for 5 min. The temperature at the injection port was 250°C. The components of the test solution were identified by comparing the spectra with those of known compounds stored in internal library.

### Bacterial isolates

A total of 36 bacterial isolates (See Table 1) from 10 different species of aquatic animals (*Penaeus vannamei*, *Penaeus monodon*, *Macrobrachium rosenbergii*, *Scylla* sp., *Rana catesbeiana*, *Lates calcarifer*, *Clarias gariepinus*, *Tilapia* sp., *Monopterus albus* and *Trichogaster pectoralis*) were applied in the present study. Seven ATCC bacterial type strains (*Aeromonas hydrophila* ATCC 49140, *Yersinia enterolitica* ATCC 23715, *Citrobacter freundii* ATCC 8090, *Edwardsiella tarda* ATCC 15947, *Escherichia coli* ATCC 25922, *Pseudomonas aeruginosa* ATCC 35032 and *Streptococcus agalatiae* ATCC 13813) were tested as well. The bacterial isolates were cultured in tryptic soy broth for 24 h at room temperature. Concentration of the bacterial culture was adjusted to 10^9^ CFU/ ml and cross-check with a Biophotometer (Eppendorf, Germany) prior to antibiotic susceptibility test.

**Table 1:** Chemical composition of *C. nardus* essential oil.

Compound	Percentage (%)
Citronellal	29.6
2,6-octadienal, 3,7-dimethyl-, (E)-	11.0
cis-2,6-dimethyl-2,6-octadiene	6.9
Propanoic acid, 2-methyl-, 3,7-dimethyl-2,6-octadienyl ester, (E)-	6.9
Caryophyllene	6.5
Citronellol	4.8
Phenol, 2-methoxy-3-(2-propenyl)-	4.5
Cyclohexane, 1-ethenyl-1-methyl-2,4-bis (1-methylethenyl)-	3.3
Limonene	2.7
2,6-octadien-1-ol, 3,7-dimethyl-,(E)-	2.4
1,6-cyclodecadiene,1-methyl-5-methylene-8-(1-methylehtyl)-, [s-(E, E)]-	2.3
Naphthalene, 1,2,3,5,6,8a-hexahydro-4,7-dimethyl-1-(1-methylethyl)-, (1S-cis)-	1.8
2,6-octadiene, 2,6-dimethyl-	1.6
Eugenol	1.5
3,7-cyclodecadiene-1-methanol, a,a,4,8-tetramethyl-, [s-(z,z)]	1.3
Cyclohexane, 1-ethenyl-1-methyl-2,4-bis(1-methylethenyl)-,[1S-(1a,2a,4a)]-	1.3
Cyclohexanemethanol, 4-ethenyl-a,a,4-trimethyl-3-(1-methylethenyl)-, [1R-(1a,3a,4a)]-	1.3
2,6-octadien-1-ol, 3,7-dimethyl-, acetate, (E)-	1.2
Naphthalene, 1,2,4a,5,6,8a-hexahydro-4,7-dimethyl-1-(1-methylethyl)-, (1a,4aa,8aa)-	1.1
Naphthalene, 1,2,3,4,4a,5,6,8a-octahydro-7-methyl-4-methylene-1-(1-methylethyl)-, (1a, 4aa, 8aa)-	0.6
a-caryophyllene	0.3
2-Furanmethanol,5-ethenyltetrahydro-a,a-5-trimethyl-, cis-	0.2
Unknown 1	0.1
Unknown 2	6.9

### Minimum inhibitory concentration (MIC) determination

The MIC values of *C. nardus* essential oil and the positive controls, kanamycin and eugenol (Analar, UK) against bacterial isolates from aquatic animals and ATCC bacterial type strains were determined through a two-fold broth micro dilution method (11, 12). For the dilution of essential oil, 0.01% methanol was used. Bacterial suspensions were loaded into the wells of microtiter plate containing a serial dilution of citronella essential oil and positive controls. Lowest concentrations of *C. nardus* essential oil and positive controls which gave no visible turbidity after 24 h incubation at room temperature were recorded as the MIC values.

## RESULTS

A total of 22 chemical compounds were identified in *C. nardus* essential oil, representing 93.1% of the detected compounds where citronellal, or 6-octenal, 3, 7-dimethyl- was the major compound (29.6%), followed by 2,6-octadienal, 3,7-dimethyl-, (E)- (11.0%), cis-2,6-dimethyl-2,6-octadiene (6.9%) and propanoic acid, 2-methyl-, 3,7-dimethyl-2,6-octadienyl ester, (E)- (6.9%) (See Table 1). In the present study, *C. nardus* essential oil was able to inhibit the growth of all 36 bacterial isolates from cultured aquatic animals as well as 7 ATCC bacterial type strains. The MIC values of *C. nardus* essential oil against the tested bacterial isolates ranged from 0.244 µg/ml to 0.977 µg/ml, whereas MIC values of kanamycin and eugenol against the tested bacterial isolates ranged from 15 µg/ml to 125 µg/ml and 15,625 µg/ml to 250,000 µg/ml, respectively (See Table 2).

**Table 2:** Minimum inhibitory concentration (MIC) values of *C. nardus* essential oil, kanamycin and eugenol against systemic bacteria isolated from cultured aquatic animals and ATCC bacterial type strains.

Bacterial species	Source	*C. nardus* essential oil (µg/ml)	Kanamycin (µg/ml)	Eugenol (µg/ml)
*Edwardsiella* spp.	*Lates calcarifer*	0.488	31	62,500
*Edwardsiella* spp.	*Macrobrachium rosenbergii*	0.488	31	62,500
*Edwardsiella* spp.	*Rana catesbeiana*	0.977	15	250,000
*Edwardsiella tarda*	*Clarias gariepinus*	0.488	15	62,500
*Edwardsiella tarda*	*Clarias gariepinus*	0.488	31	62,500
*Edwardsiella tarda*	*Clarias gariepinus*	0.488	31	62,500
*Edwardsiella tarda*	*Clarias gariepinus*	0.488	31	62,500
*Edwardsiella tarda*	*Clarias gariepinus*	0.488	31	62,500
*Edwardsiella tarda*	*Clarias gariepinus*	0.488	62	62,500
*Edwardsiella tarda*	*Clarias gariepinus*	0.977	31	125,000
*Edwardsiella tarda*	*Tilapia* sp.	0.244	31	32,500
*Edwardsiella tarda*	*Tilapia* sp.	0.244	125	15,625
*Edwardsiella tarda*	*Monopterus albus*	0.244	31	32,500
*Edwardsiella tarda*	*Monopterus albus*	0.244	31	15,625
*Edwardsiella tarda*	*Monopterus albus*	0.244	62	15,625
*Edwardsiella tarda*	*Monopterus albus*	0.244	125	32,500
*Edwardsiella tarda*	*Monopterus albus*	0.488	31	62,500
*Edwardsiella tarda*	*Trichogaster pectoralis*	0.244	31	15,625
*Edwardsiella tarda*	*Trichogaster pectoralis*	0.488	31	32,500
*Edwardsiella tarda*	*Trichogaster pectoralis*	0.488	31	62,500
*Edwardsiella tarda*	*Trichogaster pectoralis*	0.488	31	62,500
*Vibrio* spp.	*Macrobrachium rosenbergii*	0.244	62	31,250
*Vibrio* spp.	*Penaeus monodon*	0.244	31	15,625
*Vibrio* spp.	*Penaeus vannamei*	0.244	31	15,625
*Vibrio* spp.	*Rana catesbeiana*	0.244	31	31,250
*Vibrio* spp.	*Scylla* sp.	0.244	62	15,625
*Vibrio* *damsela*	*Lates calcarifer*	0.488	31	32,500
*Aeromonas* spp.	*Macrobrachium rosenbergii*	0.488	31	62,500
*Aeromonas* spp.	*Rana catesbeiana*	0.977	15	125,000
*Escherichia* *coli*	*Macrobrachium rosenbergii*	0.488	31	62,500
*Escherichia* *coli*	*Lates calcarifer*	0.488	62	62,500
*Salmonella* spp.	*Macrobrachium rosenbergii*	0.488	31	62,500
*Salmonella* spp.	*Lates calcarifer*	0.244	31	15,625
*Flavobacterium* spp.	*Rana catesbeiana*	0.977	62	125,000
*Pseudomonas* spp.	*Lates calcarifer*	0.244	31	32,500
*Streptococcus* spp.	*Lates calcarifer*	0.488	62	62,500
*Aeromonas hydrophila*	ATCC 49140	0.488	31	62,500
*Yersinia enterocolitica*	ATCC 23715	0.488	31	62,500
*Citrobacter freundii*	ATCC 8090	0.244	31	32,500
*Edwardsiella tarda*	ATCC 15947	0.244	31	32,500
*Escherichia coli*	ATCC 25922	0.244	31	32,500
*Pseudomonas aeruginosa*	ATCC 35032	0.244	31	32,500
*Streptococcus agalactiae*	ATCC13813	0.244	31	32,500

## DISCUSSION

Chemical compounds of *C. nardus* essential oil were found in different composition in related scientific reports. However, it is in agreement that citronellal is the major compound of *C. nardus* essential oil, which gives the characteristic lemongrass aroma (15). Higher percentage of citronellal (35%) was detected by Koba et al. (2009) compared to 29.6% detected in present study and 5.8% by Nakahara et al. (16). Discrepancies in the percentage of chemical compounds were also noted for citronellol in present study (4.8%), compared to Nakahara et al. (2003) (4.6%) and Koba et al. (15) (10.7%). Geraniol, or 2,6-octadienal, 3,7-dimethyl-, (E)- detected in present study (2.4%) was much lower compared to studies by Nakahara et al. (16) (35.7%), Oussalah et al. (9) (19.1%) and Koba et al. (15) (27.9%). On the other hand, 2.7% limonene found in present study was not detected in the study by Nakahara et al. (16) but present as much as 10.7% in Koba et al. (15) study.

The application of antibiotics in the treatment of bacterial diseases in fish culture is one of the greatest veterinary achievements of the past century. However, due to the gene exchange and mutation, many species of fish pathogenic bacteria were no longer sensitive to all known antibiotic. Therefore, new antimicrobial agents should be developed to minimize antibiotic resistance problems by pathogenic bacteria in aquatic environment. The efficacy of *C. nardus* essential oil as antimicrobial agents were agreeable to Hammer et al. (14), where the application of *C. nardus* essential oil inhibited various types of human pathogens such as *Acinetobacter baumanii*, *Enterococcus faecalis*, *Escherichia coli*, *Klebsiella pneumoniae*, *Pseudomonas aeruginosa*, *Salmonella typhimurium*, *Serratia marcescens* and *Staphylococcus aureus* (14) at the concentration of 1200 µg/ml to < 20000 µg/ml. However, the MIC values reported by Hammer et al. (14) were much higher compared to the MIC values of *C. nardus* essential oil in the present study. This may indicate that bacterial isolates from aquatic animals were more susceptible to *C. nardus* essential oil compared to human pathogens. The differences in chemical composition of *C. nardus* essential oil may partly due to the difference in extraction techniques, geographical sources and maturity stages of *C. nardus*. Nevertheless, compounds of *C. nardus* essential oil collectively exhibited growth inhibition effect on both Gram negative and Gram positive bacterial species tested in present study.

## CONCLUSION

This is the first report on antimicrobial property of *C. nardus* essential oil against systemic bacteria isolated from various aquatic animals. Essential oil of *C. nardus* demonstrated its potential as alternative to commercial antibacterial agent.
